# The Clinical and Cost-Effectiveness of Telerehabilitation for People With Nonspecific Chronic Low Back Pain: Randomized Controlled Trial

**DOI:** 10.2196/15375

**Published:** 2020-06-24

**Authors:** Francis Fatoye, Tadesse Gebrye, Clara Fatoye, Chidozie E Mbada, Mistura I Olaoye, Adesola C Odole, Olumide Dada

**Affiliations:** 1 Faculty of Health, Psychology and Social Care Manchester Metropolitan University Manchester United Kingdom; 2 College of Health Sciences Obafemi Awolowo University Osun Nigeria; 3 College of Medicine University of Ibadan Oyo Nigeria

**Keywords:** cost-utility analysis, quality-adjusted life years, telerehabilitation, low back pain, mobile phone

## Abstract

**Background:**

Telerehabilitation can facilitate multidisciplinary management for people with nonspecific chronic low back pain (NCLBP). It provides health care access to individuals who are physically and economically disadvantaged.

**Objective:**

This study aimed to evaluate the clinical and cost-effectiveness of telerehabilitation compared with a clinic-based intervention for people with NCLBP in Nigeria.

**Methods:**

A cost-utility analysis alongside a randomized controlled trial from a health care perspective was conducted. Patients with NCLBP were assigned to either telerehabilitation-based McKenzie therapy (TBMT) or clinic-based McKenzie therapy (CBMT). Interventions were carried out 3 times weekly for a period of 8 weeks. Patients’ level of disability was measured using the Oswestry Disability Index (ODI) at baseline, week 4, and week 8. To estimate the health-related quality of life of the patients, the ODI was mapped to the short-form six dimensions instrument to generate quality-adjusted life years (QALYs). Health care resource use and costs were assessed based on the McKenzie extension protocol in Nigeria in 2019. Descriptive and inferential data analyses were also performed to assess the clinical effectiveness of the interventions. Bootstrapping was conducted to generate the point estimate of the incremental cost-effectiveness ratio (ICER).

**Results:**

A total of 47 patients (TBMT, n=21 and CBMT, n=26), with a mean age of 47 (SD 11.6) years for telerehabilitation and 50 (SD 10.7) years for the clinic-based intervention, participated in this study. The mean cost estimates of TBMT and CBMT interventions per person were 22,200 naira (US $61.7) and 38,200 naira (US $106), respectively. QALY gained was 0.085 for TBMT and 0.084 for CBMT. The TBMT arm was associated with an additional 0.001 QALY (95% CI 0.001 to 0.002) per participant compared with the CBMT arm. Thus, the ICER showed that the TBMT arm was less costly and more effective than the CBMT arm.

**Conclusions:**

The findings of the study suggested that telerehabilitation for people with NCLBP was cost saving. Given the small number of participants in this study, further examination of effects and costs of the interventions is needed within a larger sample size. In addition, future studies are required to assess the cost-effectiveness of this intervention in the long term from the patient and societal perspective.

## Introduction

Low back pain (LBP) can result from several different abnormalities or diseases. It is commonly accompanied by pain in one or both legs, between the lower rib margins, and in the buttock creases [1]. Almost 90% and 10% cases of LBP are of nonspecific and specific causes, respectively [2]. The prevalence of LBP in those aged 9 to 18 years in high-income, medium-income, and low-income countries was around 40.0% [3]. It has also been reported that most adults will have LBP at some point during their lifetime [4]. LBP was responsible for around 60.1 million years lived with disability globally in 2015, and there will be an overall increase in its global burden because of population increase and aging [5]. The working age groups in middle-income and low-income countries have the highest disability from LBP [6]. A review of studies in the United States and internationally suggested that the costs of treating LBP are extremely high, where indirect costs represented a majority of the overall costs associated with LBP [7]. Dagenais et al [7] also indicated that the largest proportion of direct medical costs for LBP was spent on physical therapy and inpatient hospital services, followed by pharmacy and primary care. In relation to nonspecific chronic low back pain (NCLBP), there are no specific treatments that can be provided. The reason for this is that the pathoanatomical cause for nonspecific LBP is unknown [8].

Many clinical practice guidelines are recommended for the prevention and management of LBP [9]. These practice guidelines include education that supports self-management and resumption of normal activities and exercise, use of medication, imaging, and surgery. Research studies from high-income countries suggest that exercise alone, and exercise in combination with education, reduces the risks of an episode of LBP [10]. Compared with no treatment, a supervised exercise for children and adolescents can improve average pain intensity by 2.9 points (95% CI 1.6 to 4.1) in patients with LBP [11]. On the other hand, Steffens et al [10] concluded that physiotherapy interventions such as education alone, back belts, and shoe insoles did not appear to prevent LBP.

Despite the availability of many clinical guidelines for managing LBP, a substantial difference in their applicability exists in high-income as well as low-income and middle-income countries [12]. Identifying the best intervention for LBP can not only improve the health outcomes for patients but also reduce health care utilization and costs associated with the management of the condition. Telerehabilitation, in the form of a mobile phone app platform extension exercise that enables a patient to perform exercises using a smartphone, may be a practical intervention for LBP in geographically remote areas with a shortage of services and a lack of access to physical therapy rehabilitation services. Telerehabilitation uses communication technology for the remote delivery of care to patients and has the potential to manage multiple components of health, including functional independence, self-care, and self-management of illness [13].

The findings from a review of 29 articles indicated that telehealth had a moderate, positive, and significant effect on clinical outcomes for different patient populations, including LBP, heart, and psychiatric conditions [14]. In a few studies included in the systematic review, the use of telerehabilitation for patients with LBP was reported to have positive clinical outcomes, which may in turn lead to fewer visits to the emergency room and physician, fewer admission to hospitals, shorter length of stay in hospitals, and lower costs [14]. Despite the methodological differences in studies and the health care system of various countries, understanding the clinical outcomes and the economic costs of telerehabilitation interventions may improve their efficiency. The use of telerehabilitation in low- and middle-income countries such as Nigeria is just emerging; as a result, data on the clinical and cost-effectiveness of telerehabilitation are scarce [15,16]. To date, we are not aware of any study that has investigated the clinical and cost-effectiveness of physiotherapy using telerehabilitation in these countries. To study the clinical and cost-effectiveness of telerehabilitation, we developed a telerehabilitation-based McKenzie exercise intervention for people with NCLBP. This study, therefore, assessed the clinical and cost-effectiveness of telerehabilitation-based McKenzie therapy (TBMT) compared with clinic-based McKenzie therapy (CBMT) for people with NCLBP in Nigeria.

## Methods

### Trial Design

This study was an experimental research design and was conducted at the department of physiotherapy, Ladoke Akintola University of Technology University Teaching Hospital, Osogbo, and the physiotherapy department, State Hospital, Ejigbo. Ethical approval for this study was obtained from the Health Research Ethical Committee of the Institute of Public Health, Obafemi Awolowo University (registration number: IPH/OAU/12/515).

### Study Population

The sample size for this study was determined using equation 1 [17]:


m (size per group)=c×π_1_ (1−π_1_) + π_2_ (1−π_2_)/(π_1_−π_2_)^2^ (**1**)

where c=7.9 for 80% power and π_1_ and π_2_ are the proportion estimates (π_1_=0.25 and π_2_=0.65). Therefore, m=0.25 [(1−0.25) + 0.65 (1−0.65)]/(0.25−0.65)^2^=20.49, which is approximately 21. Hence, the calculated sample size was 42 (21 per group). To account for a possible attrition of 10% (ie, 4.2), the estimated minimum sample size was 46.

Patients with NCLBP, who attended outpatient physiotherapy departments, were recruited into this study. At the start of the recruitment process, the purpose of the research was explained to the participants. All participants (n=70) who were assessed for eligibility in the study were provided an informed written consent form translated by experts into the local language.

A research assistant recorded the number of participants who were invited to participate, the number of participants who declined to participate, and the number of screened patients who were not eligible and their reasons for declining participation. Eligibility for participation in this study was based on physician referral and physiotherapists’ diagnosis of NCLBP. Participants with a clinical diagnosis of long-term NCLBP aged between 20 and 65 years and those without any obvious deformities affecting the trunk or upper and lower extremities were included. The term *long-term* was used in this study instead of chronic. Using the International Classification of Functioning, Health and Disability framework, it is believed that the word *chronic* may be associated with negative expectations; therefore, the word *long-term* is preferred [18]. In addition, patients included in the study were those without any apparent deformities in the trunk and upper and lower extremities. To have a homogeneous sample of LBP type that is amenable to the McKenzie therapy, directional preference for extension was a major inclusion criterion. Directional preference is defined as the movement or posture that decreases or centralizes pain that emanates from the spine or increases the range of movement [19]. Patients with LBP who had a known comorbidity or history of cardiovascular disease for which exercise was contraindicated were excluded from this study. In addition, patients who were pregnant, those who had a previous back surgery or an experience of the McKenzie therapy, and those with directional preference for flexion or no directional preference based on the McKenzie assessment were excluded from this study.

### Randomization

A research assistant who was not involved in the assessment and treatment of the participants randomly allocated participants to the different treatment groups. The same assistant who was not involved in the assessment and treatment of the participants randomly allocated participants who volunteered to participate and satisfied the eligibility criteria to the different treatment groups (A or B). To ensure equal-sized treatment groups, random permuted blocks were used [20], and a block size of 4 was chosen (ie, AABB, ABAB, and all the other possible restricted permutations). The block permutations were computer generated using a factorial equation formula shown in equation 2:


(4!)/((2!)(2!)=24 (**2**)

The consecutive participants were randomized following the computer-generated block permutations. The printouts of all the 24 restricted computer-generated block permutation sequences were sequentially numbered, cut, and placed in a sealed envelope.

This study utilized blocked randomization because of its advantage to ensure an equal-size treatment group. Hence, this rigorous assignment method was intended to be a strength to the design of the study. However, the differences in sample size between groups were not due to random assignment but because participants declined or refused to participate, which was beyond the control of the researchers. The participants were randomly assigned to either the CBMT group or the TBMT group.

### Telerehabilitation-Based McKenzie Therapy

The TBMT group received a mobile phone–based app of Mechanical Diagnosis and Therapy (MDT). Most of the participants in the TBMT group were provided with smartphones within the available budget. Others with their own phones were recruited into that arm of the study to be able to achieve a minimum sample size, whereas those without an Android phone that could run the app were excluded.

TBMT is a comparable version of CBMT performed at home with the assistance of a mobile phone app. The mobile app is a combination of the McKenzie extension protocol and back care education developed and enabled to run on a smartphone or an Android phone with an operating system of version 3.5. TBMT is a mobile phone video app designed for patients with chronic LBP. The app incorporated personalized and guided self-therapy using the same protocol as the McKenzie protocol (ie, extension lying prone, extension in prone, and extension in standing). Performance feedback and progress tracking were telemonitored through enhanced caregiver support to improve patient engagement and therapy compliance.

### Clinic-Based McKenzie Therapy

The CBMT group received the McKenzie extension protocol and a set of back care education instructions comprising a 9-item instructional guide on standing, sitting, lifting, and other activities of daily living at home [19]. The protocol involves a course on specific lumbosacral repeated movements in extension that cause the symptoms to centralize, decrease, or abolish [21]. The extension activities include extension lying prone, extension in prone, and extension in standing repeated up to 10 times [19,21]. The determination of the directional performance for extension was followed by the extension protocol. The details of the protocol have been described in an earlier publication [22].

- Extension lying prone: participant laid prone, with elbows placed under the shoulders so that he/she could lean on the forearms, and stayed in this position for 5 min. The movement was repeated up to 10 times.
- Extension in prone: participant positioned in prone, placed his/her hands under the shoulders in the press-up position. The participant then straightened the elbows and pushed the top half of the body up as far as his/her pain permits. The participant maintained the position for up to 2 seconds. The movement was repeated up to 10 times.
- Extension in standing: participant stood upright with the feet slightly apart and placed his/her hands in the small of the back with the fingers pointing backward. The participant then stretched the trunk backward at the waist level as far as he/she can, using the hands as a fulcrum while keeping the knees straight. The movement was repeated up to 10 times.

### Outcomes and Assessment

Baseline assessment was carried out for each participant who was recruited into the study. Anthropometric variables such as weight and height were measured. Information such as age, gender, educational level, occupation, marital status, onset of back pain, recurrence, duration of complaint, and previous intervention were recorded for each participant accordingly. The participants were also assessed for directional preference. It involved repeated movements, of 5 to 10 sets of each movement, and it included movements in standing and lying positions and in sagittal and frontal planes while the participants’ symptomatic and mechanical responses were assessed. Following the repeated-movement testing, the participants returned to the same standing position, and following standardized instructions in the McKenzie Institute’s Lumbar Spine Assessment Algorithm (MILSAA), they were asked if the pain was centralizing or peripheralizing during and after movements or if there was no effect. The MILSAA is a well-defined algorithm that leads to the simple classification of spine-related disorders. This is based on a consistent *cause and effect* relationship between historical pain behavior as well as the pain response to repeated test movements, positions, and activities during the assessment process. The participants’ mechanical response to repeated movements was used to establish their directional preference.

Treatment health outcomes were assessed at 4 weeks and 8 weeks of the study, and the outcome evaluators were blinded to the groups and the interventions. A primary outcome of the LBP disability was used as a health outcome, which was measured by the Oswestry Disability Index (ODI). The ODI is a self-administered questionnaire on a 10-item scale with 6 response categories [18]. Each item scores from 0 (better) to 5 (worse). Each score was transferred into a 0 to 100 scale. The ODI score of each patient was recorded. To estimate the health-related quality of life of patients, the ODI score was mapped to short-form six dimensions (SF-6D) instrument using equation 3 [23]:


SF-6D=0.78275–0.00518 (ODI) (**3**)

The SF-6D is a preference-based health state classification system [24]. The SF-6D values obtained using the above formula were important for measuring the health outcomes of patients, and this enabled the researchers to perform a cost-utility analysis (CUA). The CUA is used to determine the cost in terms of utilities, and it combines the quantity and quality of life. An increased quality of life of LBP participants can be expressed as a utility value on a scale of 0 (dead) to 1 (perfect quality of life). After obtaining the SF-6D values of each participant, the quality-adjusted life year (QALY) of each participant was calculated. QALY was calculated by multiplying the SF-6D values and the duration of time (years). For the purpose of this study, the average of QALYs at 4 weeks and 8 weeks was considered for the participants in the study.

### Resource Use and Costs

Health care resource use and costs were assessed based on the McKenzie extension protocol, focusing on the direct implementation of costs of TBMT and CBMT. The direct health care resources included for implementation were the back-treatment DVD that was used for dummy app development before the real app was developed, development of the mobile phone–based app of the MDT for smartphones, and Android phones with an operating system of version 3.5. In addition to these, smartphones with the app installed for patients who may not have smartphones, phone credits for calls, internet data use for the entire project period, and fee for consultations were among the resources used. These resources were documented from McKenzie therapy protocols. Personal costs associated with CBMT were not included in this analysis. As the patients were those attending outpatient physiotherapy departments, the costs of medications were not included in this study. Moreover, in the context of this study, most of the patients can access health care through out-of-pocket means, in addition to undisclosed self-medication practices that are often encouraged by over-the-counter access to more than the regulated medications.

### Statistical and Cost-Effectiveness Analysis

Descriptive statistics of the mean or SD and an inferential data analysis were performed using Statistical Packages for the Social Sciences version 23 (IBM Corp). A nonparametric Mann-Whitney *U* test and Friedman test were used to compare the mean effects between the treatment regimen across the fourth- and eighth-week period and the changes of the effects of the interventions from baseline at the fourth week and eighth week for the categorical variables, respectively. A significance level of *P*=.05 was adopted for those comparisons.

The incremental cost-effectiveness ratio (ICER) was used to assess the cost-effectiveness of TBMT compared with CBMT using the formula shown in equation 4 [25]:


ICER=Δ Cost/Δ Effectiveness=(Cost of TBMT−Cost of CBMT)/(QALY for TBMT−QALY for CBMT) (**4**)

The ICER is the differential costs and outcomes between the new intervention (TBMT) and the control (CBMT). The numerator in the cost-effectiveness ratio is the monetary cost of the TBMT intervention minus the monetary cost of CBMT. The annual costs of the projects were calculated by converting the 8-week costs, the period used for implementation. The denominator is the QALY gained by TBMT minus the QALY gained by CBMT. Bootstrapping was used for a pair-wise comparison of the mean costs and effects between the TBMT and CBMT groups. CIs for the mean differences in effects were obtained by bootstrapping (1000 replications). The bootstrapped cost and effect pairs were also graphically represented on a cost-effectiveness plane [26].

## Results

### Data Source and Selection

**Figure 1:**
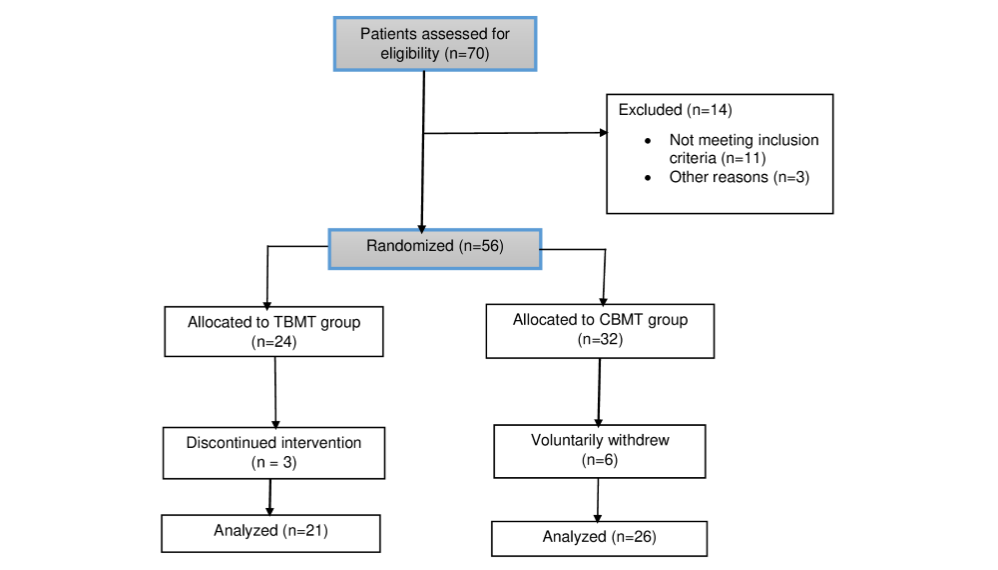
Flowchart of included patients. CBMT: clinic-based McKenzie therapy; TBMT: telerehabilitation-based McKenzie therapy.

A total of 47 participants (CBMT, n=26 and TBMT, n=21) were randomized and provided baseline data (Figure 1). Table 1 shows the baseline characteristics of these participants. The occupations of the participants were trading (n=13), teaching (n=7), nursing (n=3), tailoring (n=6), and others (n=18). The mean age of the participants was 47.3 (SD 11.6) years and 50 (SD 10.7) years for the TBMT group and CBMT group, respectively. The participants in the TBMT group had higher weight and BMI (8.1 kg and 1.5 kg/m^2^, respectively) than those in the CBMT group. A pain duration of 9.8 (SD 2.7) months was reported for the participants in the TBMT group, which was less than that of the CBMT group, a pain duration of 8.3 (SD 3.2) months. From this study, weight (kg) was the only anthropometric characteristic that was significantly different between groups at baseline. However, BMI was not statistically different between both groups. The most common causes of chronic LBP in the participants were lifting, poor posture, prolonged sitting, bending, standing, and rigorous activity.

**Table 1:** Baseline characteristics of the telerehabilitation-based McKenzie therapy group and clinic-based McKenzie therapy group.

Variables		Telerehabilitation-based McKenzie therapy group (n=21)	Clinic-based McKenzie therapy group (n=26)	*P* value
Age (years), mean (SD)		47.3 (11.6)	50.0 (10.7)	.40
Weight (kg), mean (SD)		79.1 (13.1)	71.0 (7.8)	.01
BMI (kg/m^2^), mean (SD)		27.9 (3.6)	26.4 (3.4)	.15
Height (m), mean (SD)		1.7 (0.1)	1.6 (0.1)	.11
Pain duration (months), mean (SD)		9.8 (2.7)	8.3 (3.2)	.10
**Occupation, n**				
	Trading	4	9	N/A^a^
	Teaching	2	5	N/A
	Nursing	2	1	N/A
	Tailoring	2	4	N/A
	Artisan	4	2	N/A
	Driver	0	1	N/A
	Civil service	6	4	N/A
	Student	1	0	N/A

^a^N/A: not applicable.

### Resource Use and Costs

Participants in the CBMT and TBMT groups provided the cost data (Table 2). The cost estimates for SMS messages and reminder calls were 50 naira (US $0.14) per unit, and the cost estimate of owning a compatible phone for the app was 20,000 naira (US $55.56). The resource use and costs for CBMT were as follows: cost estimate of each clinic visit (3 visits per week; 1000 naira [US $2.78] per visit) and transportation and refreshment estimate for each clinic visit (500 naira [US $1.39] per visit). Moreover, the common costs of both groups were costs of physiotherapy consultation (before randomization into the group), and they were estimated to be 1000 naira (US $2.78).

**Table 2:** Cost associated with implementation of telerehabilitation-based McKenzie therapy and clinic-based McKenzie therapy.

Resources	Cost per visit (US$)		Total cost per participant (US$)	
	TBMT^a^	CBMT^b^	TBMT	CBMT
SMS messages and reminder calls (3 times per week)	0.14	0.14	3.4	3.4
Compatible phones for the app	55.6	N/A^c^	55.6	N/A
Clinic visit (3 visits per week)	N/A	2.8	N/A	66.7
Consultation fee	2.8	2.8	2.8	2.8
Transportation and refreshment	N/A	1.4	N/A	33.4
Total cost	N/A	N/A	61.8	106.3

^a^TBMT: telerehabilitation-based McKenzie therapy.

^b^CBMT: clinic-based McKenzie therapy.

^c^N/A: not applicable.

### Effectiveness

The mean change of clinical effectiveness of CBMT and TBMT from baseline at weeks 4 and 8 is presented (Table 3). The changes of health outcomes from baseline to week 4 and week 8 have shown a significant difference (*P*<.001) within the CBMT and TBMT groups. However, no significant or clinically relevant mean ODI score difference was observed in the measurements at weeks 4 and 8 between the CBMT and TBMT groups (*P*>.05).

### Cost-Effectiveness

**Figure 2:**
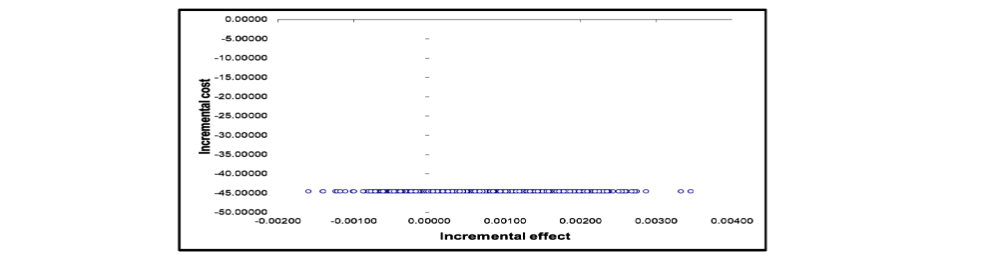
The incremental cost-effectiveness plane for a plot of 1000 bootstrap incremental costs and effects resample means. The blue circles are the plots of 1000 bootstraps incremental costs and effects resample means.

Table 4 reports the point estimates of the incremental costs and effects per patient. A reduction in the total health care cost in the participants who received TBMT was reported, 16,000 naira (US $44.26), compared with those who received CBMT. On the other hand, participants who received TBMT had an additional health benefit (0.001 QALY) compared with those who received CBMT. Thus, the ICER showed that the TBMT arm was less costly and more effective than the CBMT arm. Figure 2 shows the incremental cost-effectiveness plane for a plot of 1000 bootstrap incremental cost and effect resample means.

**Table 3:** Estimates of clinical effectiveness at weeks 4 and 8 after randomization.

Oswestry Disability Index	Mean change from baseline (95% CI)^a^		Mean treatment difference (95% CI)^b^	*P* value
	Clinic-based McKenzie therapy	Telerehabilitation-based McKenzie therapy		
Week 4	8.5 (5.45 to 11.55)	10.43 (7.74 to 11.54)	1.61 (−2.1 to 5.43)	.24
Week 8	14.50 (10.63 to 18.36)	15.71 (12.85 to 18.57)	0.81 (−2.39 to 4.01)	.58

^a^*P*<.001.

^b^The mean treatment difference suggests the comparison of the health outcomes of CBMT and TBMT at week 4 and week 8.

**Table 4:** Incremental cost-effectiveness ratio.

Intervention	Cost, naira (US$)	Incremental cost, naira (US$)	Effects, mean QALY^a^ (95% CI),	Incremental effect, mean QALY^a^ (95% CI),	Incremental cost-effectiveness ratio of naira (US$)/QALY gained
Clinic-based McKenzie therapy	38,200 (106.22)	N/A^b^	0.084 (0.084 to 0.085)	N/A	N/A
Telerehabilitation-based McKenzie therapy	22,200 (61.7)	−16,000 (−44.26)	0.085 (0.80 to 0.09)	0.001 (0.001 to 0.002)	Dominant

^a^QALY: quality-adjusted life year.

^b^N/A: not applicable.

## Discussion

### Overview

This is the first study to examine the clinical and cost-effectiveness of telerehabilitation compared with clinic-based therapy. The mean treatment effect of the participants was assessed at week 4 and week 8. A significant difference was found for clinical effectiveness within the TBMT and CBMT groups from baseline to week 4 and week 8. On the other hand, no significant difference of the mean ODI score was reported between the two intervention groups. The findings of this study are in line with the results of the study by Kosterink et al [27], who investigated the effects of a 4-week teletreatment service in subjects with nonspecific neck and shoulder pain, where they showed that the treatment was effective in reducing pain intensity and disability over time. They also reported that there was no significant difference between teletreatment and conventional care—where subjects did not receive any specific intervention such as osteopathy, chiropractice, ergonomic counseling, medication, physiotherapy, acupuncture, stress management, and relaxation training.

In line with the study conducted in Norway on patients with musculoskeletal problems, the results of this study indicated that telerehabilitation therapy was cost saving [28]. Also, it is understood that both cost and health benefits of the two interventions could have an impact on the cost-effectiveness of telerehabilitation. This study showed that telerehabilitation was less costly than clinic-based treatment. In line with our study, a cost analysis study in Canada, which was conducted on patients with a knee problem, also concluded that the cost of telerehabilitation was lower than that of conventional rehabilitation [29]. Moreover, a cost-minimization study on patients with skeletal problems in Finland has also indicated that telemedicine was less costly for society than conventional care at a workload of more than 80 patients per year [30].

The increment or reduction of the costs and effectiveness of the TBMT by half from the base case values was unlikely to affect its cost-effectiveness in this study. The findings of this study are consistent with the results of the cost-effectiveness analysis study on telemedicine for primary care delivery, where telemedicine was shown to be cost saving as long as its effectiveness was greater than that of the controlled intervention [28]. However, the reduction of the health benefits from the base case values in this study could lead telerehabilitation not to be a cost-effective intervention. Overall, it is important that patients adhere to telerehabilitation services and improve their health for the new intervention to be cost-effective.

TBMT was approximately 50% cheaper than CBMT; this is because of less requirements of a clinic-based facility and less contact with a physiotherapist for its delivery. In other words, there is an opportunity to implement telerehabilitation programs across numerous geographic locations if needed. In low-income countries, such as Nigeria, access to physiotherapy services is a challenge because of the shortage of physiotherapists and limited access to clinic-based programs [31]. Unlike CBMT, TBMT could overcome barriers to accessing physiotherapy services and could provide numerous benefits with reduced cost to the patients in Nigeria. However, the key challenges for its implementation strategies are the existence of effective internet services and patient reluctance to engage [32].

### Strengths and Limitations

The major strength of this study was that it is the first study in low- and middle-income countries to evaluate the cost-effectiveness of telerehabilitation therapy for patients with NCLBP using a randomized controlled trial. In addition, the findings of this study could inform clinicians and decision makers about the implementation of TBMT as a complementary option of CBMT services in Nigeria. On the other hand, the findings reported here should be viewed in the context of the limitations of this study. The cost analysis did not include costs of medications and indirect costs. It is believed that the exclusion of costs of medications and indirect costs to the cost-effectiveness analysis may underestimate the total cost of therapies. The second limitation of the study was related to the time of follow-up; the effects of the telerehabilitation therapies might be different in the long-term follow-up. Thus, evidence of health benefits from a long-term follow-up of patients is important to be incorporated in the cost-effectiveness analysis of telerehabilitation.

### Conclusions

The findings of this study showed that telerehabilitation was associated with greater health benefits and lower costs, suggesting that it was a cost-saving therapy compared with a clinic-based therapy. This suggests that the implementation of TBMT could help to overcome barriers to access to physiotherapy services, particularly in low-income countries such as Nigeria, thereby improving the health outcomes of patients in these countries. Future studies are required to assess the cost-effectiveness of the intervention in the long term from the patient and societal perspective.

## Appendix

Multimedia Appendix 1 CONSORT-EHEALTH checklist (V 1.6.1).

## Abbreviations

CBMT: clinic-based McKenzie therapy
CUA: cost-utility analysis
ICER: incremental cost-effectiveness ratio
LBP: low back pain
MDT: Mechanical Diagnosis and Therapy
MILSAA: McKenzie Institute’s Lumbar Spine Assessment Algorithm
NCLBP: nonspecific chronic low back pain
ODI: Oswestry Disability Index
QALY: quality-adjusted life year
SF-6D: short-form six dimensions
TBMT: telerehabilitation-based McKenzie therapy
