# Supplementary material for: The Clinical and Cost-Effectiveness of Telerehabilitation for People With Nonspecific Chronic Low Back Pain: Randomized Controlled Trial
Source: JMIR Mhealth Uhealth. 2020 Jun 24;8(6):e15375. doi: 10.2196/15375 (PMC7381065; doi:10.2196/15375)
Supplement: Multimedia Appendix 1 [file mhealth_v8i6e15375_app1.pdf]

CONSORT-EHEALTH Checklist V1.6.2 Report

Manuscript Number 15375

(based on CONSORT-EHEALTH V1.6), available at [http://tinyurl.com/consort-ehealth-v1-6].

Date completed

6/10/2020 13:07:42

by

Tadesse Gebrye

TITLE

1a-i) Identify the mode of delivery in the title

1a-ii) Non-web-based components or important co-interventions in title

ABSTRACT

Background: Telerehabilitation can facilitate multidisciplinary management for people with nonspecific chronic low back pain (NCLBP). It provides health care access to individuals who are physically and economically disadvantaged.

Objective: This study aimed to evaluate the clinical and cost-effectiveness of telerehabilitation compared with a clinic-based intervention for people with NCLBP in Nigeria.

1a-iii) Primary condition or target group in the title

ABSTRACT

1b-i) Key features/functionalities/components of the intervention and comparator in the METHODS section of the ABSTRACT

1b-ii) Level of human involvement in the METHODS section of the ABSTRACT

'Methods: A cost-utility analysis alongside a randomized controlled trial from a health care perspective was conducted. Patients with NCLBP were assigned to either telerehabilitation-based McKenzie therapy (TBMT) or clinic-based McKenzie therapy (CBMT). Interventions were carried out 3 times weekly for a period of 8 weeks. Patients' level of disability was measured using the Oswestry Disability Index (ODI) at baseline, week 4, and week 8. To estimate the health-related quality of life of the patients, the ODI was mapped to the short-form six dimensions instrument to generate quality-adjusted life years (QALYs). Health care resource use and costs were assessed based on the McKenzie extension protocol in Nigeria in 2019. Descriptive and inferential data analyses were also performed to assess the clinical effectiveness of the interventions. Bootstrapping was conducted to generate the point estimate of the incremental cost-effectiveness ratio (ICER)."

1b-iii) Open vs. closed, web-based (self-assessment) vs. face-to-face assessments in the METHODS section of the ABSTRACT

1b-iv) RESULTS section in abstract must contain use data

1b-v) CONCLUSIONS/DISCUSSION in abstract for negative trials

INTRODUCTION

2a-i) Problem and the type of system/solution

2a-ii) Scientific background, rationale: What is known about the (type of) system

'Low back pain (LBP) can result from several different abnormalities or diseases. It is commonly accompanied by pain in one or both legs, between the lower rib margins, and in the buttock creases . Almost 90% and 10% cases of LBP are of nonspecific and specific causes, respectively. The prevalence of LBP in those aged 9 to 18 years in high-income, medium-income, and low-income countries was around 40.0%. It has also been reported that most adults will have LBP at some point during their lifetime. LBP was responsible for around 60.1 million years lived with disability globally in 2015, and there will be an overall increase in its global burden because of population increase and aging. The working age groups in middle-income and low-income countries have the highest disability from LBP. A review of studies in the United States and internationally suggested that the costs of treating LBP are extremely high, where indirect costs represented a majority of the overall costs associated with LBP. Dagenais et al also indicated that the largest proportion of direct medical costs for LBP was spent on physical therapy and inpatient hospital services, followed by pharmacy and primary care. In relation to nonspecific chronic low back pain (NCLBP), there are no specific treatments that can be provided. The reason for this is that the pathoanatomical cause for nonspecific LBP is unknown."

METHODS

3a) CONSORT: Description of trial design (such as parallel, factorial) including allocation ratio

3b) CONSORT: Important changes to methods after trial commencement (such as eligibility criteria), with reasons

Despite the methodological differences in studies and the health care system of various countries, understanding the clinical outcomes and the economic costs of telerehabilitation interventions may improve their efficiency. The use of telerehabilitation in low- and middle-income countries such as Nigeria is just emerging; as a result, data on the clinical and cost-effectiveness of telerehabilitation are scarce. To date, we are not aware of any study that has investigated the clinical and cost-effectiveness of physiotherapy using telerehabilitation in these countries. To study the clinical and cost-effectiveness of telerehabilitation, we developed a telerehabilitation-based McKenzie exercise intervention for people with NCLBP. This study, therefore, assessed the clinical and cost-effectiveness of telerehabilitation-based McKenzie therapy (TBMT) compared with clinic-based McKenzie therapy (CBMT) for people with NCLBP in Nigeria.

3b-i) Bug fixes, Downtimes, Content Changes

4a) CONSORT: Eligibility criteria for participants

This study was an experimental research design and was conducted at the department of physiotherapy, Ladoke Akintola University of Technology University Teaching Hospital, Osogbo, and the physiotherapy department, State Hospital, Ejigbo. Ethical approval for this study was obtained from the Health Research Ethical Committee of the Institute of Public Health, Obafemi Awolowo University (registration number: IPH/OAU/12/515).

4a-i) Computer / Internet literacy

4a-ii) Open vs. closed, web-based vs. face-to-face assessments:

4a-iii) Information giving during recruitment

'Participants were consecutively recruited but randomly assigned to the two treatment groups"

4b) CONSORT: Settings and locations where the data were collected

A research assistant recorded the number of participants who were invited to participate, the number of participants who declined to participate, and the number of screened patients who were not eligible and their reasons for declining participation. Eligibility for participation in this study was based on physician referral and physiotherapists' diagnosis of NCLBP. Participants with a clinical diagnosis of long-term NCLBP aged between 20 and 65 years and those without any obvious deformities affecting the trunk or upper and lower extremities were included. The term long-term was used in this study instead of chronic. Using the International Classification of Functioning, Health and Disability framework, it is believed that the word chronic may be associated with negative expectations; therefore, the word long-term is preferred [18]. In addition, patients included in the study were those without any apparent deformities in the trunk and upper and lower extremities. To have a homogeneous sample of LBP type that is amenable to the McKenzie therapy, directional preference for extension was a major inclusion criterion. Directional preference is defined as the movement or posture that decreases or centralizes pain that emanates from the spine or increases the range of movement [19]. Patients with LBP who had a known comorbidity or history of cardiovascular disease for which exercise was contraindicated were excluded from this study. In addition, patients who were pregnant, those who had a previous back surgery or an experience of the McKenzie therapy, and those with directional preference for flexion or no directional preference based on the McKenzie assessment were excluded from this study.		
**4b-i) Report if outcomes were (self-)assessed through online questionnaires**		
**4b-ii) Report how institutional affiliations are displayed**		
'A primary outcome of the LBP disability was used as a health outcome, which was measured by the Oswestry Disability Index (ODI). The ODI is a self-administered questionnaire on a 10-item scale with 6 response categories."		
**5) CONSORT: Describe the interventions for each group with sufficient details to allow replication, including how and when they were actually administered**		
**5-i) Mention names, credential, affiliations of the developers, sponsors, and owners**		
**5-ii) Describe the history/development process**		
**5-iii) Revisions and updating**		
**5-iv) Quality assurance methods**		
**5-v) Ensure replicability by publishing the source code, and/or providing screenshots/screen-capture video, and/or providing flowcharts of the algorithms used**		
**5-vi) Digital preservation**		
**5-vii) Access**		
**5-viii) Mode of delivery, features/functionalities/components of the intervention and comparator, and the theoretical framework**		
'Clinic"		
**5-ix) Describe use parameters**		
'The comparator (CBMT) group received the McKenzie extension protocol and a set of back care education instructions comprising a 9-item instructional guide on standing, sitting, lifting, and other activities of daily living at home. The protocol involves a course on specific lumbosacral repeated movements in extension that cause the symptoms to centralize, decrease, or abolish. The extension activities include extension lying prone, extension in prone, and extension in standing repeated up to 10 times. The determination of the directional performance for extension was followed by the extension protocol. The details of the protocol have been described in an earlier publication".		
**5-x) Clarify the level of human involvement**		
**5-xi) Report any prompts/reminders used**		
**5-xii) Describe any co-interventions (incl. training/support)**		
'Participants in the CBMT and TBMT groups provided the cost data (Table 2). The cost estimates for SMS messages and reminder calls"		
**6a) CONSORT: Completely defined pre-specified primary and secondary outcome measures, including how and when they were assessed**		
' Eligibility for participation in this study was based on physician referral and physiotherapists' diagnosis of NCLBP. Participants with a clinical diagnosis of long-term NCLBP aged between 20 and 65 years and those without any obvious deformities affecting the trunk or upper and lower extremities were included. The term long-term was used in this study instead of chronic. Using the International Classification of Functioning, Health and Disability framework, it is believed that the word chronic may be associated with negative expectations; therefore, the word long-term is preferred. In addition, patients included in the study were those without any apparent deformities in the trunk and upper and lower extremities. To have a homogeneous sample of LBP type that is amenable to the McKenzie therapy, directional preference for extension was a major inclusion criterion. Directional preference is defined as the movement or posture that decreases or centralizes pain that emanates from the spine or increases the range of movement. Patients with LBP who had a known comorbidity or history of cardiovascular disease for which exercise was contraindicated were excluded from this study. In addition, patients who were pregnant, those who had a previous back surgery or an experience of the McKenzie therapy, and those with directional preference for flexion or no directional preference based on the McKenzie assessment were excluded from this study."		
**6a-i) Online questionnaires: describe if they were validated for online use and apply CHERRIES items to describe how the questionnaires were designed/deployed**		
**6a-ii) Describe whether and how "use" (including intensity of use/dosage) was defined/measured/monitored**		
**6a-iii) Describe whether, how, and when qualitative feedback from participants was obtained**		
**6b) CONSORT: Any changes to trial outcomes after the trial commenced, with reasons**		
'This study was an experimental research design and was conducted at the department of physiotherapy, Ladoke Akintola University of Technology University Teaching Hospital, Osogbo, and the physiotherapy department, State Hospital, Ejigbo"		
**7a) CONSORT: How sample size was determined**		
**7a-i) Describe whether and how expected attrition was taken into account when calculating the sample size**		
**7b) CONSORT: When applicable, explanation of any interim analyses and stopping guidelines**		
'Baseline assessment was carried out for each participant who was recruited into the study. Anthropometric variables such as weight and height were measured. Information such as age, gender, educational level, occupation, marital status, onset of back pain, recurrence, duration of complaint, and previous intervention were recorded for each participant accordingly. The participants were also assessed for directional preference. It involved repeated movements, of 5 to 10 sets of each movement, and it included movements in standing and lying positions and in sagittal and frontal planes while the participants' symptomatic and mechanical responses were assessed. Following the repeated-movement testing, the participants returned to the same standing position, and following standardized instructions in the McKenzie Institute's Lumbar Spine Assessment Algorithm (MILSAA), they were asked if the pain was centralizing or peripheralizing during and after movements or if there was no effect. The MILSAA is a well-defined algorithm that leads to the simple classification of spine-related disorders. This is based on a consistent cause and effect relationship between historical pain behavior as well as the pain response to repeated test movements, positions, and activities during the assessment process. The participants' mechanical response to repeated movements was used to establish their directional preference.<br><br>Treatment health outcomes were assessed at 4 weeks and 8 weeks of the study, and the outcome evaluators were blinded to the groups and the interventions. A primary outcome of the LBP disability was used as a health outcome, which was measured by the Oswestry Disability Index (ODI). The ODI is a self-administered questionnaire on a 10-item scale with 6 response categories [18]. Each item scores from 0 (better) to 5 (worse). Each score was transferred into a 0 to 100 scale. The ODI score of each patient was recorded. "		
**8a) CONSORT: Method used to generate the random allocation sequence**		

'The mean change of clinical effectiveness of CBMT and TBMT from baseline at weeks 4 and 8 is presented (Table 3). The changes of health outcomes from baseline to week 4 and week 8 have shown a significant difference ($P<.001$) within the CBMT and TBMT groups. However, no significant or clinically relevant mean ODI score difference was observed in the measurements at weeks 4 and 8 between the CBMT and TBMT groups ($P>.05$)."

8b) CONSORT: Type of randomisation; details of any restriction (such as blocking and block size)

No

9) CONSORT: Mechanism used to implement the random allocation sequence (such as sequentially numbered containers), describing any steps taken to conceal the sequence until interventions were assigned

'The sample size for this study was determined using equation 1 [17]:

$m \text{ (size per group)} = c \times \pi_1 (1-\pi_1) + \pi_2 (1-\pi_2)/(\pi_1-\pi_2)^2$ (1)

where $c=7.9$ for 80% power and π_1 and π_2 are the proportion estimates ($\pi_1=0.25$ and $\pi_2=0.65$). Therefore, $m=0.25 [(1-0.25) + 0.65 (1-0.65)]/(0.25-0.65)^2=20.49$, which is approximately 21. Hence, the calculated sample size was 42 (21 per group). To account for a possible attrition of 10% (ie, 4.2), the estimated minimum sample size was 46.

Patients with NCLBP, who attended outpatient physiotherapy departments, were recruited into this study."

10) CONSORT: Who generated the random allocation sequence, who enrolled participants, and who assigned participants to interventions

No

11a) CONSORT: Blinding - If done, who was blinded after assignment to interventions (for example, participants, care providers, those assessing outcomes) and how

11a-i) Specify who was blinded, and who wasn't

11a-ii) Discuss e.g., whether participants knew which intervention was the "intervention of interest" and which one was the "comparator"

'Treatment health outcomes were assessed at 4 weeks and 8 weeks of the study, and the outcome evaluators were blinded to the groups and the interventions."

11b) CONSORT: If relevant, description of the similarity of interventions

No

12a) CONSORT: Statistical methods used to compare groups for primary and secondary outcomes

Not applicable

12a-i) Imputation techniques to deal with attrition / missing values

12b) CONSORT: Methods for additional analyses, such as subgroup analyses and adjusted analyses

Not applicable

RESULTS

13a) CONSORT: For each group, the numbers of participants who were randomly assigned, received intended treatment, and were analysed for the primary outcome

13b) CONSORT: For each group, losses and exclusions after randomisation, together with reasons

'Not applicable"

13b-i) Attrition diagram

14a) CONSORT: Dates defining the periods of recruitment and follow-up

'A total of 47 participants (CBMT, n=26 and TBMT, n=21) were randomized and provided baseline data (Figure 1). Table 1 shows the baseline characteristics of these participants. The occupations of the participants were trading (n=13), teaching (n=7), nursing (n=3), tailoring (n=6), and others (n=18). The mean age of the participants was 47.3 (SD 11.6) years and 50 (SD 10.7) years for the TBMT group and CBMT group, respectively. The participants in the TBMT group had higher weight and BMI (8.1 kg and 1.5 kg/m^2, respectively) than those in the CBMT group. A pain duration of 9.8 (SD 2.7) months was reported for the participants in the TBMT group, which was less than that of the CBMT group, a pain duration of 8.3 (SD 3.2) months. From this study, weight (kg) was the only anthropometric characteristic that was significantly different between groups at baseline. However, BMI was not statistically different between both groups. The most common causes of chronic LBP in the participants were lifting, poor posture, prolonged sitting, bending, standing, and rigorous activity."

14a-i) Indicate if critical "secular events" fell into the study period

14b) CONSORT: Why the trial ended or was stopped (early)

'Not applicable"

15) CONSORT: A table showing baseline demographic and clinical characteristics for each group

No

15-i) Report demographics associated with digital divide issues

16a) CONSORT: For each group, number of participants (denominator) included in each analysis and whether the analysis was by original assigned groups

16-i) Report multiple "denominators" and provide definitions

16-ii) Primary analysis should be intent-to-treat

No

17a) CONSORT: For each primary and secondary outcome, results for each group, and the estimated effect size and its precision (such as 95% confidence interval)

No

17a-i) Presentation of process outcomes such as metrics of use and intensity of use

17b) CONSORT: For binary outcomes, presentation of both absolute and relative effect sizes is recommended

Baseline characteristics of the telerehabilitation-based McKenzie therapy group and clinic-based McKenzie therapy group.

VariablesTelerehabilitation-based McKenzie therapy group (n=21)Clinic-based McKenzie therapy group (n=26)P value
Age (years), mean (SD)47.3 (11.6)50.0 (10.7).40
Weight (kg), mean (SD)79.1 (13.1)71.0 (7.8).01
BMI (kg/m^2), mean (SD)27.9 (3.6)26.4 (3.4).15
Height (m), mean (SD)1.7 (0.1)1.6 (0.1).11
Pain duration (months), mean (SD)9.8 (2.7)8.3 (3.2).10

18) CONSORT: Results of any other analyses performed, including subgroup analyses and adjusted analyses, distinguishing pre-specified from exploratory

Estimates of clinical effectiveness at weeks 4 and 8 after randomization.<br><br>Oswestry Disability IndexMean change from baseline (95% CI)aMean treatment difference (95% CI)bP value<br><br>Clinic-based McKenzie therapyTelerehabilitation-based McKenzie therapy<br><br>Week 48.5 (5.45 to 11.55)10.43 (7.74 to 11.54)1.61 (−2.1 to 5.43).24<br>Week 814.50 (10.63 to 18.36)15.71 (12.85 to 18.57)0.81 (−2.39 to 4.01).58<br><br>a P<.001.<br><br>bThe mean treatment difference suggests the comparison of the health outcomes of CBMT and TBMT at week 4 and week 8.		
**18-i) Subgroup analysis of comparing only users**		
**19) CONSORT: All important harms or unintended effects in each group**		
No		
**19-i) Include privacy breaches, technical problems**		
**19-ii) Include qualitative feedback from participants or observations from staff/researchers**		
DISCUSSION		
**20) CONSORT: Trial limitations, addressing sources of potential bias, imprecision, multiplicity of analyses**		
**20-i) Typical limitations in ehealth trials**		
**21) CONSORT: Generalisability (external validity, applicability) of the trial findings**		
**21-i) Generalizability to other populations**		
**21-ii) Discuss if there were elements in the RCT that would be different in a routine application setting**		
**22) CONSORT: Interpretation consistent with results, balancing benefits and harms, and considering other relevant evidence**		
**22-i) Restate study questions and summarize the answers suggested by the data, starting with primary outcomes and process outcomes (use)**		
**22-ii) Highlight unanswered new questions, suggest future research**		
This is the first study to examine the clinical and cost-effectiveness of telerehabilitation compared with clinic-based therapy. The mean treatment effect of the participants was assessed at week 4 and week 8. A significant difference was found for clinical effectiveness within the TBMT and CBMT groups from baseline to week 4 and week 8. On the other hand, no significant difference of the mean ODI score was reported between the two intervention groups. The findings of this study are in line with the results of the study by Kosterink et al [27], who investigated the effects of a 4-week teletreatment service in subjects with nonspecific neck and shoulder pain, where they showed that the treatment was effective in reducing pain intensity and disability over time. They also reported that there was no significant difference between teletreatment and conventional care—where subjects did not receive any specific intervention such as osteopathy, chiropractice, ergonomic counseling, medication, physiotherapy, acupuncture, stress management, and relaxation training.		
Other information		
**23) CONSORT: Registration number and name of trial registry**		
No		
**24) CONSORT: Where the full trial protocol can be accessed, if available**		
No		
**25) CONSORT: Sources of funding and other support (such as supply of drugs), role of funders**		
Registration number: IPH/OAU/12/515		
**X26-i) Comment on ethics committee approval**		
**x26-ii) Outline informed consent procedures**		
**X26-iii) Safety and security procedures**		
**X27-i) State the relation of the study team towards the system being evaluated**		
